# Metabolomic Profile of Primary Turkey and Rat Hepatocytes and Two Cell Lines after Chloramphenicol Exposure

**DOI:** 10.3390/ani10010030

**Published:** 2019-12-21

**Authors:** Lidia Radko, Tomasz Śniegocki, Bartosz Sell, Andrzej Posyniak

**Affiliations:** Department of Pharmacology and Toxicology, National Veterinary Research Institute, Al. Partyzantów 57, 24-100 Puławy, Poland; sniego@piwet.pulawy.pl (T.Ś.); bartosz.sell@piwet.pulawy.pl (B.S.); aposyniak@piwet.pulawy.pl (A.P.)

**Keywords:** primary turkey hepatocytes, primary rat hepatocytes, HepG2 cell line, Balb/c 3T3 cell line, chloramphenicol, metabolites, HPLC-MS/MS method, cytotoxicity

## Abstract

**Simple Summary:**

The use of cell cultures can be an important alternative for animal experiments. Therefore, it seems reasonable to use in vitro models to compare the liver metabolism of a drug in different animal species. Chloramphenicol is an effective broad-spectrum antibiotic used in human and pets. The toxicity of chloramphenicol is complex and differs among animal species mainly depending on the biotransformation. The purpose of this study was to assess chloramphenicol metabolism on its cytotoxicity in primary turkey and rat hepatocyte cultures, also in human hepatoma (HepG2) cells and nonhepatic, Balb/c 3T3 fibroblasts. To the best of our knowledge, this is the first report of differences in chloramphenicol metabolism in primary turkey and rat hepatocyte cultures. The two metabolites of the drug were detected in turkey and rat hepatocyte cultures. The amount of one metabolite of chloramphenicol was closely related to the cytotoxicity of the drug. The primary turkey and rat hepatocyte cultures were more sensitive to chloramphenicol than HepG2 cells and Balb/c 3T3 fibroblasts. The primary hepatocyte cultures represent valuable tools with which to study the biotransformation of xenobiotics and determine species differences in their metabolism and toxicity.

**Abstract:**

The purpose of this study was to assess the formation of chloramphenicol metabolites in primary turkey and rat hepatocyte cultures and human hepatoma (HepG2) cells and nonhepatic, Balb/c 3T3 fibroblasts. Additionally, the cytotoxicity of the drug was assessed through three biochemical endpoints: mitochondrial and lysosomal activity and cellular membrane integrity after 24 and 48 h exposure. The two metabolites of the drug, chloramphenicol glucuronide and nitroso-chloramphenicol, were detected to the greatest extent in both primary hepatocyte cultures by liquid chromatography–tandem mass spectrometry. Toxic nitroso-chloramphenicol was the main metabolite in the primary turkey hepatocyte cultures, but it was not in the primary rat hepatocyte cultures. The most affected endpoint in turkey and rat hepatocyte cultures was the disintegration of the cellular membrane, but in the cell lines, mitochondrial and lysosomal activities underwent the greatest change. The primary hepatocyte cultures represent valuable tools with which to study the species differences in the biotransformation and toxicity of drugs. To the best of our knowledge, this is the first report of differences in chloramphenicol metabolism in primary turkey and rat hepatocyte cultures.

## 1. Introduction

Chloramphenicol (CAP) is a broad-spectrum antibiotic used in medicine. Its use in food-producing animals is banned due to the potentially carcinogenic action of CAP residue and the development of non-dose-related aplastic anemia in humans [1,2]. However, this antibiotic is used illegally in veterinary practice, and its residues are found in food from animals (milk (0.005–0.5 µg/L), honey (0.1–75 µg/kg), eggs (0.9 µg/kg), fish and shrimp (0.01–242 µg/kg), meat (0.004–0.01 µg/kg), turkey breasts (1–8.7 µg/kg), and chicken breasts (range, 0.4–1.2 µg/kg)) [3,4,5] and the environment (sediments (0.196 mg/kg), surface water (112 ng/kg)) [6,7,8,9]. CAP is rapidly absorbed following oral administration and distributed throughout the organs and tissues in animals and humans. The use of CAP in humans can lead to serious hematologic and metabolic toxicity (e.g., “gray baby syndrome” in newborns and young infants) [1,2]. The drug is metabolized primarily in the liver [10] and its use by patients with liver cancer may pose a risk of adverse drug reaction [11]. Additionally, the drug shows toxicity to the liver and reproductive system in animals [2]. The mechanism of CAP’s toxicity is complex and differs from species to species, depending mainly on how CAP undergoes biotransformation [1,12]. According to the European Food Safety Authority (EFSA), research on CAP should focus on the potential formation of reactive (toxic) metabolites of the antibiotic in different animal species, which may result in the presence of residues in food of animal origin [2]. To our knowledge there is no information about metabolites of CAP in turkeys. The data on CAP kinetics are scant in avian species, and only one report is available for the turkey [13].

Metabolomics can provide appraisal of a biological system’s biochemical and physiological status, e.g., with cell culture analysis [14,15]. Of all omics technologies, metabolomics is thought to best represent “classical toxicology” [16]. Metabolome analyses not only serve very well to gain us more information from studies, but they also help to reduce animal testing by refining the methods. Here, we combined mass-spectrometry metabolomics with an in vitro study.

Nowadays, numerous studies use in vitro experimental models in order to reduce animal experimentation because in vivo modelling faces consequential ethical, social, law, and economic challenges. Assuming that toxic effects seen in a whole organism are due to prior failure of basic cellular functions, cytotoxicity studies offer a good source of information about the potential mechanism of action [17,18,19,20,21]. However, it is necessary to remember that the results depend on many factors such as the nature of compound, nature of assay, model used, and time of exposure. Because no single test is able to gain reliable information about mechanism of cytotoxicity, the best way is to use a battery of tests that evaluate different endpoints. For this reason, lactate dehydrogenase (LDH) leakage, neutral red uptake (NRU), and 3-(4,5-dimethylthiazol-2-yl)-2,5-diphenyl tetrazolium bromide (MTT) reduction assays are the most frequently used. The LDH is a stable cytoplasmic enzyme present in all cells. It is rapidly released from cells upon damage of the plasma membrane. The MTT assay is based on the reduction of tetrazolium salt MTT in live cells to dark formazan product inside the cell via mitochondrial nicotinamide adenine dinucleotide phosphate (NADPH)-dependent dehydrogenase. The amount of formazan generated is assumed to be directly proportional to the cell number. The neutral red assay is based on staining living cells by neutral red dye, which readily diffuses through the plasma membrane and concentrates in lysosomes. The screening of drugs for a particular adverse property currently is the most applicable use of in vitro tests. Knowledge of metabolism is of great importance in the area of drug safety because biotransformation can lead either to detoxification or to formation of more toxic metabolites. Since the liver is the main target organ in systemic toxicity, and since it plays a major role in the metabolism of many compounds, the liver-derived cell models are among the most frequently used in the in vitro studies. Therefore, it seems reasonable to use in vitro models to compare the liver metabolism of a drug in different animal species. Primary cultures of hepatocytes and liver-derived lines are the models that are used for the evaluation of drugs of which toxicity is mediated through biotransformation [22,23]. Because of the capacity to maintain a sufficient level of xenobiotic metabolism, primary cultures of hepatocytes from poultry and mammals were used in this study. The primary rat hepatocyte cultures are widely used for toxicological and metabolic studies and are well standardized [24,25]. The intensive breeding of food production animals and their diet (with phytoestrogen content and addition of hormones or antibiotics to feed) results in low viability of isolated hepatocytes. Therefore, the turkeys from which the hepatocytes in this study were isolated were bred in laboratory-controlled conditions. The difficult standardization and the scarce availability and difficult logistics of human liver cells prevent their larger-scale use. The human hepatoma (HepG2) cell line was well characterized for its usefulness for examining the cytotoxicity of the substances affecting cell metabolism [26,27,28,29]. The HepG2 cells were selected as target cells for evaluating toxicity to human liver cancer and have been extensively used as the test system for the prediction of toxicity and metabolites in cancer patients [30]. The toxic effect of CAP metabolites was verified in a metabolically inactive cell model, Balb/3T3 fibroblasts. Balb/c 3T3 is the cell line most frequently used to screen the general toxicity of chemicals and is recommended by the Organization for Economic Co-operation and Development (OECD) in a guidance document 129 [31].

The objective of this research was to investigate CAP metabolite formation in different cell cultures. In addition, the effects of biotransformation of CAP on its cytotoxicity were assayed. The concentration range of the drug was chosen according to its plasma (5–15 µg/mL in most animal species) and residue levels in animal products and the environment [1,2,3,4,5,6,7,8]. The study was performed on primary turkey and rat hepatocyte cultures, human hepatoma (HepG2) cells, and nonhepatic Balb/c 3T3 fibroblasts. Hepatocytes were used to mimic optimal metabolism in healthy turkey and rat organisms and to indicate differences between these species. The human hepatoma cells can serve as an indicator of the metabolism and cytotoxic potential of CAP in cancer cells, and fibroblasts represent nonmetabolizing cells. To the best of our knowledge, it is the first report on the differences in metabolic activity between the primary culture of hepatocytes from poultry and mammals as well as cancer cells following exposure to CAP.

## 2. Materials and Methods

### 2.1. Chemicals and Reagents

Analytical standards of chloramphenicol (CAP, CAS 56-75-7), 2-isopropanol, acetic acid, deuterated internal standard (CAP-d5), dimethyl sulfoxide (DMSO), fetal bovine serum (FBS), bovine calf serum (BCS), bovine serum albumin, 3-(4.5-dimethylthiazol-2-yl)-2.5-diphenyl tetrazolium bromide (MTT), neutral red dye, Triton X-100, trypsin-EDTA, collagenase type IV, insulin, hydrocortisone, and antibiotic solutions (penicillin and streptomycin) were purchased from Sigma-Aldrich, Poznań, Poland. Chloramphenicol glucuronide (CAP-G, CAS 39751-33-2) was obtained from Toronto Research Chemicals (Toronto, ON, Canada). All other chemicals were sourced from commercial suppliers and were of the highest available purity.

### 2.2. Compound Preparation and Exposure

CAP was dissolved in DMSO, of which the final concentration was 0.1% in the tested serum- and antibiotic-free medium. The same final concentrations of the solvent and 1% Triton X-100 solution were used as negative and positive controls, respectively. CAP was tested in eight concentrations ranging from 1.56 to 200 µg/mL, and each concentration was tested in six replicates in three independent experiments. The cytotoxicity was assessed after 24 and 48 h exposure and incubation, which passed without the medium being changed.

### 2.3. Cellular Models

#### 2.3.1. The Primary Turkey Hepatocyte Cultures

##### Animals

The hepatocyte isolation procedure was carried out according to bioethical principles and with the permission of the Local Ethical Commission at the University of Life Sciences in Lublin, Poland (16/2016). Ten meat turkeys from a highly selected line (British United Turkeys Big 6) were purchased as fourteen-day-old poults from a commercial grow-out farm (Indykpol, Frednowy, Poland). Briefly, the turkey poults were housed in standard laboratory conditions on wood shavings in a pen with humidity of 50%–60% and an initial light regime of 15 h per day. After 14 d, the light regime was reduced to 10 h. The temperature within the pen was 24 °C in the first week and slowly reduced to 20 °C by week 6. An infrared heater set at 34 °C was installed to heat the resting area. All birds were fed a commercial antibiotic-free pellet diet (Agropol, Motycz, Poland) using a phase feeding regimen consisting of starter feed (DKA starter) for weeks 1 to 6 then grower feed (DKA grower) from weeks 7 to 10. Feed and water were provided ad libitum.

##### Turkey Hepatocytes Isolation and Culture Conditions

Turkey hepatocytes were isolated from birds of 4.0–6.0 kg body weight and 8–10 weeks old with a two-step collagenase perfusion technique under laboratory conditions [32]. After the turkey was anesthetized with sodium pentobarbital (10 mg/kg) (Exagon vet (Richter Pharma AG, Austria)) and anticoagulated with sodium heparin (1750 U/kg) (Heparinum (Polfa, Warsaw, Poland)), the liver was perfused with prewarmed calcium-free liver perfusion medium via the portal vein, then the inferior vena cava was cut to remove the buffer liquid from the liver. At first, perfusion was maintained at 20 to 30 mL per minute with 500–700 mL of calcium free liver perfusion medium until the liver became blanched. In the next step, 200 mL of digestion buffer with 0.05 mg/mL of collagenase type IV and 0.06 mg/L calcium chloride in calcium-free liver perfusion medium was used until the liver became soft. After the perfusion, cells were dispersed in Willam’s E medium (Gibco, Thermo Scientific, Waltham, MA, USA) with 2 mg/mL bovine serum albumin, 100 IU/mL penicillin, and 100 µg/mL streptomycin. The cells were filtered through sterilized gauze of 150 and 75 µm pore sizes to eliminate cell aggregate, centrifuged at 40× *g* for 5 min, and washed three times with the medium previously described. Then, 4 × 10^6^ cells/well were seeded into 96-well culture plates coated with collagen I (Corning BioCoat, Corning Inc., Tewksbury, MA, USA) and incubated in William’s E medium with 20% FBS, 1 nM hydrocortisone, 100 IU/mL penicillin, 100 µg/mL streptomycin, and 1 mg/mL bovine insulin in a humidified atmosphere with 5% CO_2_ at 37 °C. Following a 4 h incubation, the cells were washed with prewarmed phosphate-buffered saline (PBS). Next, the cells were incubated in William’s E medium with 1% FBS, 1 nM hydrocortisone, 1 mg/mL bovine insulin, and solutions of CAP.

#### 2.3.2. Rat Hepatocyte Isolation and Primary Hepatocyte Culture Conditions

The procedure of hepatocyte isolation was carried out according to the bioethical principles and with the permission of the Local Ethical Commission at the University of Life Sciences in Lublin, Poland (11/2015) and was described in our previous articles [24,25]. The cells were seeded on 96-well plates coated with collagen I (Corning BioCoat) at the density of 5 × 10^5^ cells/well in 100 µL of medium and were incubated until attached. After 4–5 h, the medium was replaced with fresh medium containing CAP solutions.

#### 2.3.3. Cell Lines and Culture Conditions

HepG2 cell line was purchased from the American Type Culture Collection (ATCC HB-8065) and cultured in Eagle’s Minimum Essential Medium (EMEM) (ATCC). Balb/c 3T3 clone A31 cell line (donated by the Department of Swine Diseases of the National Veterinary Research Institute in Puławy, Poland) was cultured in Dulbecco’s Modified Eagle’s Medium (DMEM) (ATCC). The culture conditions were described in our previous articles [24,25].

### 2.4. Determination of CAP and Its Metabolized Products by HPLC-MS/MS

Following 24 and 48 h incubation, the medium from the cultures was collected for evaluation of biotransformation of the drug. CAP, CAP-G, and nitroso-chloramphenicol (NO-CAP) were determined using liquid chromatography–tandem mass spectrometry (HPLC-MS/MS). The HPLC-MS/MS system consisted of an ABSciex Exion LC HPLC system connected to ABSciex API 5500 Qtrap mass spectrometer (AB Sciex, Concord, ON, Canada). The Analyst 1.6.3 software controlled the HPLC-MS/MS system, and Multiquant 3.2 was used to process the data. The MS system was operated in the electrospray negative ionization mode with a capillary voltage of 4.5 kV. The multiplier was set at 1900 V. The desolvation temperature was set at 500 °C, collision gas (N2)—high; nebuliser gas (N2)—36 psi; gas 1 (air)—35 psi; gas 2 (air)—35 psi; curtain gas (N2)—36 psi. The chromatography was performed on a Kinetex C8 column (Kinetex, C8 75 × 2.1 mm, 2.6 micron particle diameter) (Phenomenex International, Torrance, CA, USA) connected to a C8 precolumn (4 mm × 2 mm × 4 m). Identification of CAP and CAP-G in medium was performed by comparing the mass spectra from standards with the mass spectra from analyzed samples, but identification of NO-CAP was based on molecular ions and characteristic ions in analyzed samples. A method previously described was employed with some modifications [3]. The culture medium (50 µL) was diluted with 1950 µL of 0.5% isopropanol in 0.1% acetic acid, added to a vial with 50 µL of internal standard solution (CAP-d5), and injected into a chromatography column (Kinetex, C8 75 × 2.1 mm, 2.6 micron particle diameter) (Phenomenex International, Torrance, CA, USA). The mobile phase used consisted of 0.5% isopropanol in 0.1% acetic acid (A) and 5% isopropanol in ethanol (B) and it was pumped through in the gradient mode. The mobile phase composition (A : B, v : v) was started at 85 : 15 from 0 to 2.5 min, then was 55 : 45 at 3.0 min, and was held at 85 : 15 from 4.2 min to 10.0 min for re-equilibration. The column was operated at 40 °C at a flow rate of 0.4 mL/min.

Mass spectrometry analysis was performed using electrospray (ESI, negative ionization) in the multiple reaction monitoring (MRM) mode. For each analyte, two fragmentation reactions were monitored, whereas one was monitored for internal standards (CAP m/z = 321.0 → 152.0; 321.0 → 194.0, CAP-G m/z = 496.3 → 152.0; 496.3 → 321.0, NO-CAP m/z = 304.8 → 269.0; and 304.8 → 194.0, internal standard CAP-d5 m/z = 326.0→157.0). The limit of quantification (LOQ) for all analytes was 0.1 ng/mL.

Standard calibration curves were prepared by injecting standard solutions of CAP for determination of CAP and standard solutions of CAP glucuronide for determination CAP-G and NO-CAP at seven concentration levels in the 0.1–200 µg/mL range. The ratios of peak areas of standards and the internal standard were plotted versus concentration expressed as ng/mL. The equations and regression coefficients were calculated for the curves and were used to calculate the concentration of analytes. Such calibration curves were prepared for each series of samples. The concentrations of analytes were calculated as the differences between tested samples and the control sample containing CAP. 

The procedure was validated according to the (Guidance Document) SANTE/11945/2015 [33]. The validation parameters linearity, repeatability, reproducibility, average recovery, selectivity, limit of detection (LOD), and limit of quantification (LOQ) were evaluated.

### 2.5. Cytotoxicity Assessment

#### 2.5.1. LDH Assay

The method is based on the assessment of cell membrane damage by measuring the optical density of LDH released into the cellular medium [34]. The assay was performed using a commercially available cytotoxicity detection kit (LDH) (Roche Diagnostics, Warsaw, Poland) according to the manufacturer’s protocol.

#### 2.5.2. MTT Assay

The method is based on reduction of MTT by succinate dehydrogenase to the purple formazan product inside living cells [35]. The amount of formazan generated was proven to be directly proportional to the cell density. The description of the test procedure was given in a previous article [24].

#### 2.5.3. NRU Assay

The method is based on staining living cells with neutral red, which readily diffuses through the plasma membrane and accumulates in lysosomes [36]. The description of the test procedure was also given in a previous article [25].

### 2.6. Statistical Analysis

The study was performed in three independent experiments. The obtained results are presented as mean values ± SD (standard deviation). Statistical analysis was performed using GraphPad Prism 5 software (GraphPad, San Diego, CA, USA). The differences between the two means derived from the HPLC-MS/MS analysis were tested using Student’s *t*-test. Assessment of the cytotoxicity data used a non-parametric ANOVA test with Dunn’s post-hoc multiple comparison test to determine significance relative to the negative control. The cytotoxicity concentration (CC_20_) necessary for 20% inhibition of cell viability by the drug was calculated by GraphPad Prism 5.0 and expressed as mean ± SEM (standard error of mean). Statistical comparisons among CC_20_ values were performed by the analysis of variance (ANOVA) followed by the Tukey test, and differences were considered statistically significant at *p* ≤ 0.05. Pearson’s linear correlation coefficient (R) was used to assess relationships between the variables.

## 3. Results

### 3.1. Primary Turkey and Rat Hepatocyte Cultures

#### 3.1.1. Concentrations of CAP and Its Metabolites in the Cell Medium

**Figure 1:**
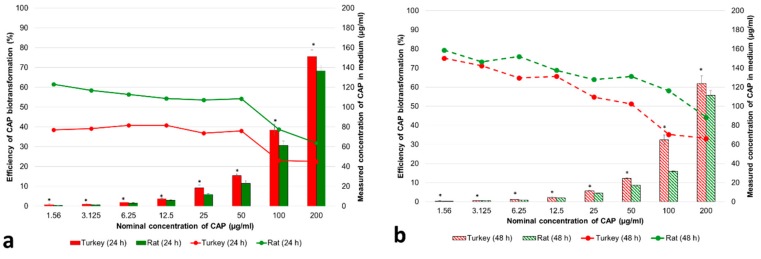
Efficiency of chloramphenicol (CAP) biotransformation and its concentrations (µg/mL) in medium with turkey and rat primary hepatocyte cultures after 24 h (**a**) and 48 h (**b**) exposure to CAP. The values are expressed as means ± SD (n = 3). The differences (* *p* ≤ 0.05) between means were analyzed using Student’s *t*-test: turkey vs. rat at 24 h (**a**) and turkey vs. rat at 48 h (**b**).

Exposure of the study cell cultures to CAP resulted in a dose-dependent increase of the concentration of the drug in medium (Figure 1a,b). CAP was metabolized efficiently by the primary turkey and rat hepatocyte cultures, and the percentage of the drug metabolized by turkey and rat hepatocytes increased with the time of exposure (Figure 1a,b). CAP metabolites were lower in turkey hepatocyte cultures than in rat hepatocyte cultures (Figure 1a,b), and the concentrations of unmetabolized CAP in turkey hepatocyte cultures were higher than in rat hepatocyte cultures at both time points (Figure 1a,b). A decrease of the concentration of the drug in medium at 48 h after turkey and rat hepatocyte cultures began to be exposed was noted from the concentration at the 24 h interval (Figure 1a,b).

**Figure 2:**
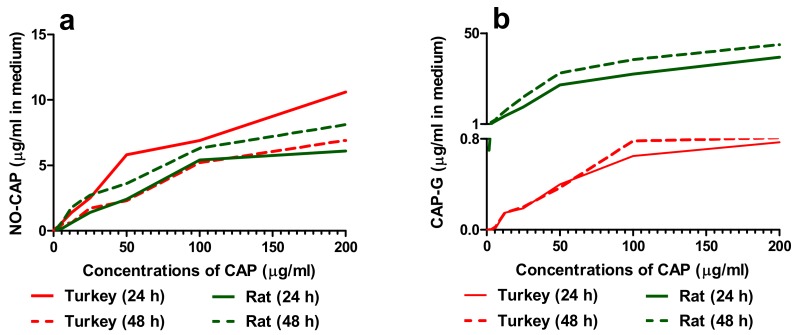
Concentrations of nitroso-chloramphenicol (NO-CAP) (**a**) and chloramphenicol glucuronide (CAP-G) (**b**) (µg/mL) in medium with primary turkey and rat hepatocyte cultures after 24 and 48 h exposure to CAP.

Two metabolites of CAP, NO-CAP, and CAP–G, were detected in turkey and rat hepatocyte cultures (Figure 2a,b).

The concentrations of both CAP metabolites in turkey and rat hepatocyte cultures in medium increased in a dose-dependent manner (Figure 2a,b). In the case of the primary turkey hepatocyte cultures, NO-CAP was the main metabolite of CAP, and its amount gradually decreased towards the end of 48 h exposure (Figure 2a). The concentrations of NO-CAP were ten times higher than those of CAP-G in the medium (Figure 2a,b). On the other hand, concentrations of CAP-G in turkey hepatocyte cultures were thirty times lower than in the rat counterparts (Figure 2b); CAP-G was the main metabolite of CAP in the mammalian hepatocyte cultures (Figure 2b). The concentrations of this metabolite were four times higher than that of NO-CAP in the medium (Figure 2a,b). The concentrations of both CAP metabolites in rat hepatocyte cultures in medium also increased in a time-dependent manner (Figure 2a,b). Examples of chromatograms for CAP and its two metabolites are shown in Supplementary Figure S1. MS^2^ spectra of CAP (a), CAP-G (b), and NO-CAP (c) are shown in Supplementary Figures S2–S4, respectively.

#### 3.1.2. Cytotoxicity

**Figure 3:**
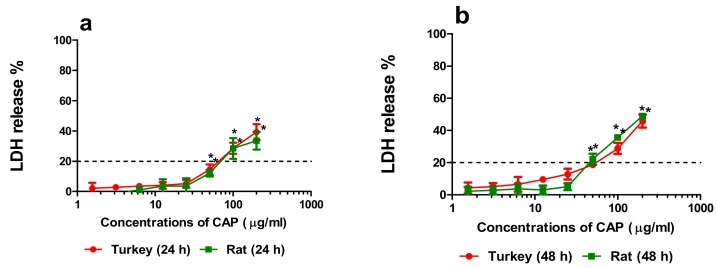
Effects of 24 h (**a**) and 48 h (**b**) incubation of primary turkey and rat hepatocyte cultures with CAP on disintegration of the cellular membrane determined by the lactate dehydrogenase (LDH) assay (mean ± SD) (n = 3). Statistical significance was evaluated by ANOVA and Dunn’s post-hoc test (* *p* ≤ 0.05).

Three commonly used cytotoxicity assays were performed that measured relevant biochemical endpoints, membrane integrity (LDH assay) as well as mitochondrial (MTT assay) and lysosomal (NRU assay) activity, after 24 h and 48 h exposure (Supplementary Figure S5). The primary turkey and rat hepatocyte cultures were the most sensitive to CAP action. Advanced membrane disintegration of turkey and rat hepatocytes was observed at a concentration of 50 µg/mL of CAP after 24 and 48 h exposure (Figure 3a,b). These effects above 20 % to an extent, were observed at 100 µg/mL of the drug (Figure 3a,b).

The minimal cytotoxicity concentration (CC_20_) values for CAP on primary turkey and rat hepatocyte cultures were only calculated for the LDH assay (Table 1). The turkey hepatocyte culture value was lower than that of rat hepatocyte cultures measured after 24 h. However, after 48 h, the reverse was true (Table 1).

#### 3.1.3. Correlation of the Quantity of NO-CAP and CAP-G in Medium with LDH Release, MTT Reduction, and NR Uptake in the Primary Hepatocytes

**Figure 4:**
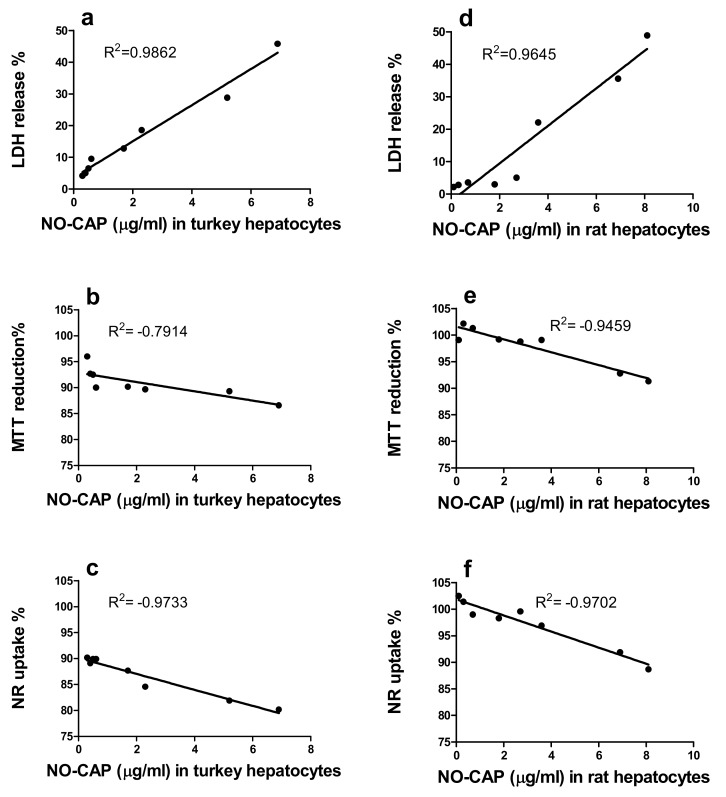
Relationship between percent of LDH released, MTT reduction, NR uptake, and concentrations of NO-CAP (µg/mL) determined in medium from primary turkey (**a**–**c**) and rat (**d**–**f**) hepatocyte cultures after exposure to CAP for 48 h.

Analysis was made of whether the LDH release, MTT reduction, and NR uptake in primary turkey and rat hepatocyte cultures correlated with the concentrations of NO-CAP in culture medium. Our results indicate a strong, positive correlation (Pearson’s *r* = 0.99 (turkey) and *r* = 0.96 (rat)) between concentrations of the metabolite and LDH release from turkey and rat hepatocyte cultures. A negative correlation in MTT reduction and NR uptake was observed (Figure 4). A similar correlation was observed between concentrations of CAP-G and LDH, MTT, and NRU assays from turkey and rat hepatocytes cultures (Supplementary Figure S7).

### 3.2. Human Hepatoma (HepG2) Cell and Balb/c 3T3 Fibroblast Cultures

#### 3.2.1. Concentrations of CAP and Its Metabolites in the Cell Medium

**Figure 5:**
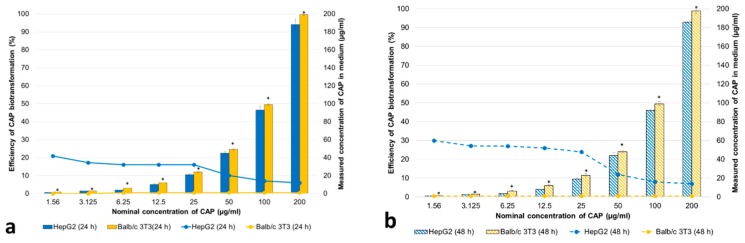
Efficiency of CAP biotransformation and its concentrations (µg/mL) in medium with HepG2 cells and Balb/c 3T3 fibroblasts after 24 h (**a**) and 48 h (**b**) exposure to the drug. The values are expressed as means ± SD (n = 3). The differences (* *p* ≤ 0.05) between means were analyzed using Student’s *t*-test: HepG2 vs. Balb/c 3T3 at 24 h (**a**) and HepG2 vs. Balb/c 3T3 at 48 h (**b**).

Exposure of HepG2 cells and Balb/c 3T3 fibroblasts to CAP resulted in a dose-dependent increase of the concentration of the drug in medium (Figure 5a,b).

CAP was poorly metabolized by HepG2 cells (Figure 5a,b). The percentage of the drug metabolized by these cells only increasing slightly with the time of exposure (Figure 5a,b), and only trace amounts of CAP-G (<LOQ) were found in the culture medium. The concentrations of the drug in Balb/c 3T3 fibroblast cultures were higher than that in HepG2 cell cultures at both time points (Figure 5a,b). The drug was not metabolized by Balb/c 3T3 fibroblasts (Figure 5a,b). Exposure of the fibroblasts to CAP did not result in change of the concentration of the drug in medium, in contrast to the clear change after HepG2 cell culture exposure (Figure 5a,b). No metabolites were found in the culture medium of Balb/c 3T3 fibroblasts (>LOQ (0.1 ng/mL)). Examples of chromatograms for CAP and its two metabolites are shown in Supplementary Figure S1.

#### 3.2.2. Cytotoxicity

**Figure 6:**
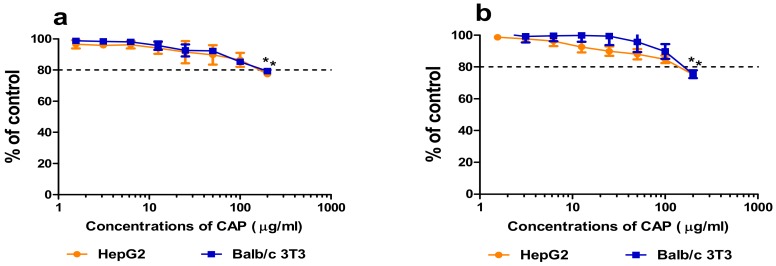
Effects of 48 h incubation of HepG2 cells and Balb/c3T3 fibroblasts with CAP on mitochondrial (**a**) and lysosomal (**b**) activity determined by the MTT and NRU assays, respectively. Results were calculated as percent of solvent control (mean ± SD) (n = 3). Statistical significance was evaluated by ANOVA and Dunn’s post-hoc tests (* *p* ≤ 0.05).

The effects of 24 and 48 h exposure to growing concentrations of CAP on membrane integrity (LDH assay), mitochondrial activity (MTT assay), and lysosomal activity (NRU assay) are shown in Supplementary Figure S6. The drug significantly inhibited cellular metabolism and lysosomal activity of HepG2 cells and Balb/c 3T3 fibroblasts at a concentration of 200 µg/mL (Figure 6a,b). After 48 h exposition, the mitochondrial and lysosomal activity of the cells decreased to below 20% at a concentration 200 µg/mL of CAP (Figure 6a,b).

The minimal cytotoxic concentration (CC_20_) values of CAP on HepG2 cells and Balb/c 3T3 fibroblasts were only calculated for the MTT and NRU assays after 48 h exposure. Based on CC_20_ values, no statistically significant differences (*p* ≤ 0.05) between tested cell lines were observed (Table 2).

## 4. Discussion

Involvement of CAP in the etiology of some diseases in humans was demonstrated using a variety of cell cultures [37,38,39]. We found that the action of the drug was dependent on the cell cultures used. The differences in the sensitivity to CAP and the mechanism of action are connected with the nitroreduction capability of individual cell types and may be critical in determining the toxicity of the drug [10,39,40]. The most important metabolite implicated in the toxicological action of CAP is NO-CAP [10]. The main product formed by glucuronidation of the drug in human and animal livers is CAP-G, which is biologically inactive [41]. These two metabolites were detected in primary turkey and rat hepatocyte cultures in this study, and trace amounts of CAP-G (<LOQ (0.1 ng/mL) were present under analysis in HepG2 cells.

The metabolomic profile varied between the primary turkey and rat hepatocyte cultures in respect to the quality and quantity of CAP metabolites formed. This is very interesting because CAP metabolism in primary turkey hepatocyte cultures has never been studied. The in vivo studies with poultry indicate that CAP metabolism differs from that of rats in the formation and excretion of glucuronide and reduction of the nitro group [2,42]. We found that the concentrations of the nontoxic metabolite, CAP-G, in turkey hepatocyte cultures were lower than those in equivalent rat hepatocyte cultures. The main route of CAP metabolism in rat hepatocytes was glucuronidation leading to formation of CAP-G. The turkey has a low liver glucuronosyltransferase level [43], and glucuronide formation as a pathway for biotransformation of CAP seems to be insignificant in comparison to the rat. It is noteworthy that the main metabolite in turkey hepatocyte cultures was toxic NO-CAP. The toxic effect of this metabolite reduced the ability of hepatocytes to metabolize the drug. Therefore, the metabolism of CAP in turkey hepatocyte cultures was slower compared to rat hepatocyte cultures. The concentrations of NO-CAP in turkey and rat hepatocyte cultures had a strong, positive correlation with cytotoxicity of the drug associated with damage to the cellular membrane of these cells. The high NO-CAP level caused cellular membrane damage in the primary hepatocyte cultures [44], and this mechanism of the drug’s action was also reported in primary human osteoblasts (IC_20_ = 60–80 µg/mL) [37]. The cytotoxicity of CAP with regard to cellular membrane disintegration was examined on protozoan *Tetrahymena pyriformis*. The results suggested that the drug with its hydrophobic-free form has the ability to partition the lipid bilayer of the cell membrane, and the membrane damage thus caused leads to cytotoxic effects [45]. Additionally, the concentrations of NO-CAP in turkey and rat hepatocyte cultures had a negative correlation with cytotoxicity of the drug associated with reduction mitochondrial and lysosomal activity. The impairment of mitochondrial energetics by inhibition of mitochondrial protein synthesis in primary human osteoblasts was observed [36]. This confirmed that NO-CAP formed in the liver is an important metabolite implicated in the toxicological action of CAP [10]. An increase in the concentration of the toxic metabolite has strongly affected the correlation between CAP-G and cytotoxicity. We found that the concentrations of the nontoxic metabolite, CAP-G, in turkey and rat hepatocyte cultures correlated with cytotoxicity of CAP. No erroneous conclusions can be made based on this correlation. It should be noted that, at the same time, an increase in the concentrations of the toxic (NO-CAP) and nontoxic metabolite (CAP-G) was observed in turkey and rat hepatocyte cultures. Based on research, NO-CAP toxicity is proven, and lack CAP-G toxicity is also proven [10,46]. It should also be noted that the potential mechanism of NO-CAP action is the generation of nitrogen radicals, which are especially toxic to cells. It is possible that CAP metabolism has overall detrimental effects on liver cells. The contribution of other CAP liver metabolites (i.e., CAP-sulphate, CAP base, CAP-alcohol, CAP-aldehyde, CAP-oxamylchloride, and CAP-oxamic acid) that were not detected by HPLC-MS/MS (< LOQ (0.1 ng/mL)) cannot be excluded when considering the toxicity of the drug [10,46].

We found that human hepatoma (HepG2) cells and Balb/c 3T3 fibroblasts were less susceptible to the cytotoxic effect of CAP than the primary turkey or rat hepatocyte cultures. Only trace amounts of nontoxic CAP-G were detected in the HepG2 cell cultures. This result may confirm the kinetics of the drug in patients with liver cancer [11]. No metabolites were found in the Balb/c 3T3 fibroblast cultures. Low or completely lacking activity of the enzymes needed for the conversion of the drug to more active or toxic metabolites may partly explain this observation. The HepG2 cells show only 10% of the P450-dependent mono-oxygenase activity of freshly isolated human adult hepatocytes [47]. Westerink and Schoonen (2007) showed that HepG2 cells have low levels of cytochromes (CYPs) but normal levels of phase II enzymes with the exception of UDP-glucuronosyltransferases [48]. Likewise, the V79 cells were less sensitive to CAP cytotoxicity than the primary rat and human hepatocyte cultures [40]. A variety of data is available in the literature concerning the inhibitory effects of CAP on various enzymes leading to decreases in mitochondrial activity [37,49]. The effect of the drug on mitochondrial activity was also shown in the studies performed on MD63 and HeLa cells [37] and RL-34 cells [49]. We found that incubation of HepG2 cells and Balb/c 3T3 fibroblasts with CAP led to inhibited mitochondrial and lysosomal activity in these cells. There were no differences in the toxic action of CAP between the studied cell lines.

## 5. Conclusions

The obtained data indicate that the primary turkey and rat hepatocyte cultures intensively metabolized CAP, which was the opposite of human hepatoma (HepG2) cells. The use in the study of the primary hepatocyte cultures made it potentially possible to determine species differences in CAP biotransformation that are closely related to its cytotoxicity. The primary turkey hepatocyte cultures slowly metabolized the drug to toxic NO-CAP, of which the concentration decreased depending on the incubation time, in contrast to rat hepatocyte cultures. The primary hepatocyte cultures represent valuable tools with which to study the biotransformation of xenobiotics and determine species differences in their metabolism and toxicity.

## Figures and Tables

**Table 1:** Mean cytotoxicity concentration (CC_20_; ± SEM; µg/mL) for CAP in primary turkey (PTH) and rat (PRH) hepatocyte cultures (n = 3) evaluated by LDH, 3-(4,5-dimethylthiazol-2-yl)-2,5-diphenyl tetrazolium bromide (MTT), and neutral red uptake (NRU) assays at 24 and 48 h of incubation.

	LDH		MTT		NRU	
	24 h	48 h	24 h	48 h	24 h	48 h
PTH	67.0 ± 3.4 ^a^	54.1 ± 2.4 ^a^	ne	ne	ne	ne
PRH	81.3 ± 3.6 ^b^	47.5 ± 1.9 ^b^	ne	ne	ne	ne

The different letters (a, b) within columns indicate significant differences (*p* ≤ 0.05) between the cell cultures at the corresponding time of exposure. Ne—no effect (CC_20_) over 200 µg/mL (the highest concentration tested).

**Table 2:** Mean CC_20_ (±SEM) (µg/mL) for CAP evaluated by LDH, MTT, and NRU assays at 24 and 48 h of incubation of HepG2 cells and Balb/c 3T3 fibroblast cultures (n = 3).

	LDH		MTT		NRU	
	24 h	48 h	24 h	48 h	24 h	48 h
HepG2	ne	ne	ne	169 ± 13.0 ^a^	ne	152 ± 4.3 ^a^
Balb/c3T3	ne	ne	ne	182 ± 13.5 ^a^	ne	168 ± 13.0 ^a^

The different letters (a, b) within columns indicate significant differences (*p* ≤ 0.05) between the cell cultures at the corresponding time of exposure. ne—no effect (CC_20_) over 200 µg/mL (the highest concentration tested).

## Supplementary Materials

The following are available online at https://www.mdpi.com/2076-2615/10/1/30/s1, Figure S1: Examples of chromatograms for CAP and its two metabolites: CAP-G and NO-CAP after exposure of primary turkey and rat hepatocyte cultures and HepG2 and Balb/c 3T3 cells to CAP at the concentration level of 100 µg/mL after 48 h; Figure S2: MS^2^ spectra of CAP; Figure S3: MS^2^ spectra of CAP-G; Figure S4: MS^2^ spectra of NO-CAP; Figure S5: Effects of CAP on the viability of turkey and rat primary hepatocytes determined by the LDH (a,d), MTT (b,d), and NRU (c,f) assays. Results were calculated as % of control (mean ± SD) (n = 3). Statistical significance was evaluated by ANOVA and Dunn’s post-hoc test (* *p* ≤ 0.05; *** *p* ≤ 0.001); Figure S6. Effects of CAP on the viability of HepG2 and Balb/c 3T3 cells determined by the LDH (a,d), MTT (b,d), and NRU (c,f) assays. Results were calculated as % of control (mean ± SD) (n = 3). Statistical significance was evaluated by ANOVA and Dunn’s post-hoc test (* *p* ≤ 0.05; *** *p* ≤ 0.001); Figure S7: Relationship between percent of LDH released, MTT reduction, NR uptake, and concentrations of CAP-G (µg/mL) determined in medium from primary turkey (a,b,c) and rat (d,e,f) hepatocyte cultures after exposure to CAP for 48 h.
