# Supplementary material for: Metabolomic Profile of Primary Turkey and Rat Hepatocytes and Two Cell Lines after Chloramphenicol Exposure
Source: Animals (Basel). 2019 Dec 21;10(1):30. doi: 10.3390/ani10010030 (PMC7022860; doi:10.3390/ani10010030)
Supplement: Supplementary file 1 [file animals-10-00030-s001.zip › Supplementary Materials/Supplementary Materials contents.docx]

Supplementary Materials: Metabolomic profile of primary turkey and rat hepatocytes and two cell lines after chloramphenicol exposure

Lidia Radko, Tomasz Śniegocki, Bartosz Sell and Andrzej Posyniak

**Contents:**

Figure S1. Examples of chromatograms for CAP and its two metabolites CAP-G and NO-CAP after exposure of primary culture of turkey and rat hepatocytes HepG2, and Balb/c 3T3 cells to CAP at the concentration level of 100 µg/ml after 48 h.

**Figure S2:** MS^2^ spectra of CAP.

**Figure S3:** MS^2^ spectra of CAP-G.

**Figure S4:** MS^2^ spectra of NO-CAP**.**

**Figure S5.** Effects of CAP on the viability of turkey and rat primary hepatocytes determined by the LDH (a, d), MTT (b, d), and NRU (c, f) assays. Triton X–100 was used as the positive control. Results are shown as mean ± SD (n = 3). Statistical significance was evaluated by ANOVA and Dunn’s post-hoc test (*p ≤ 0.05; ***p ≤ 0.001)

**Figure S6.** Effects of CAP on the viability of HepG2 and Balb/c 3T3 cells determined by the LDH (a, d), MTT (b, d), and NRU (c, f) assays. Triton X–100 was used as the positive control. Results were shown as mean ± SD (n = 3). Statistical significance was evaluated by ANOVA and Dunn’s post-hoc test (*p ≤ 0.05; ***p ≤ 0.001)

**Figure S7**. Relationship between % of LDH released, MTT reduction, NR uptake and concentrations of CAP-G (µg/ml) determined in medium from primary turkey (a, b, c) and rat (d, e, f) hepatocyte cultures after exposure to CAP for 48 h.
