# Supplementary figures and images for: Metabolomic Profile of Primary Turkey and Rat Hepatocytes and Two Cell Lines after Chloramphenicol Exposure
Source: Animals (Basel). 2019 Dec 21;10(1):30. doi: 10.3390/ani10010030 (PMC7022860; doi:10.3390/ani10010030)

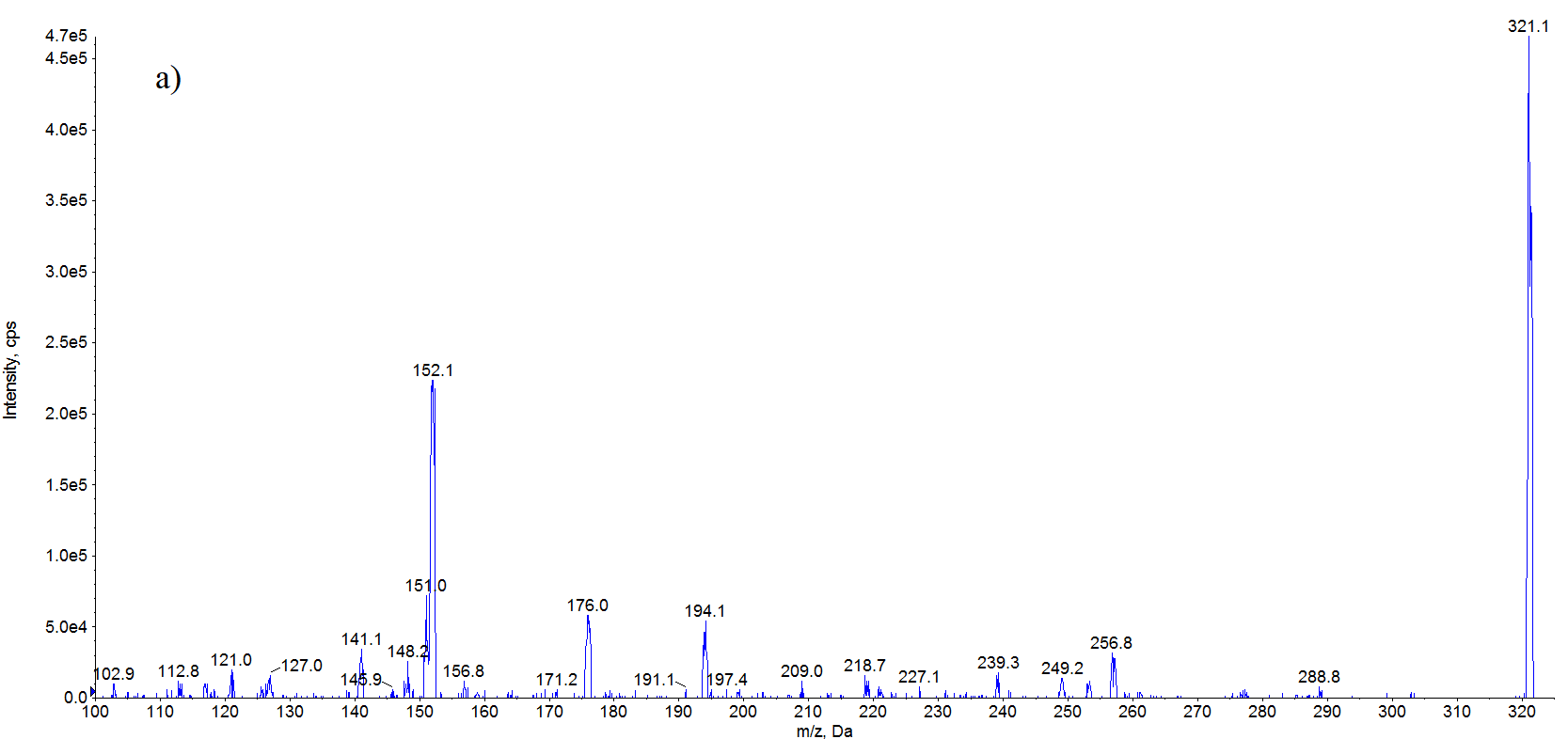

Supplement: Supplementary file 1 [file animals-10-00030-s001.zip › Supplementary Materials/Supplement 2.tif]

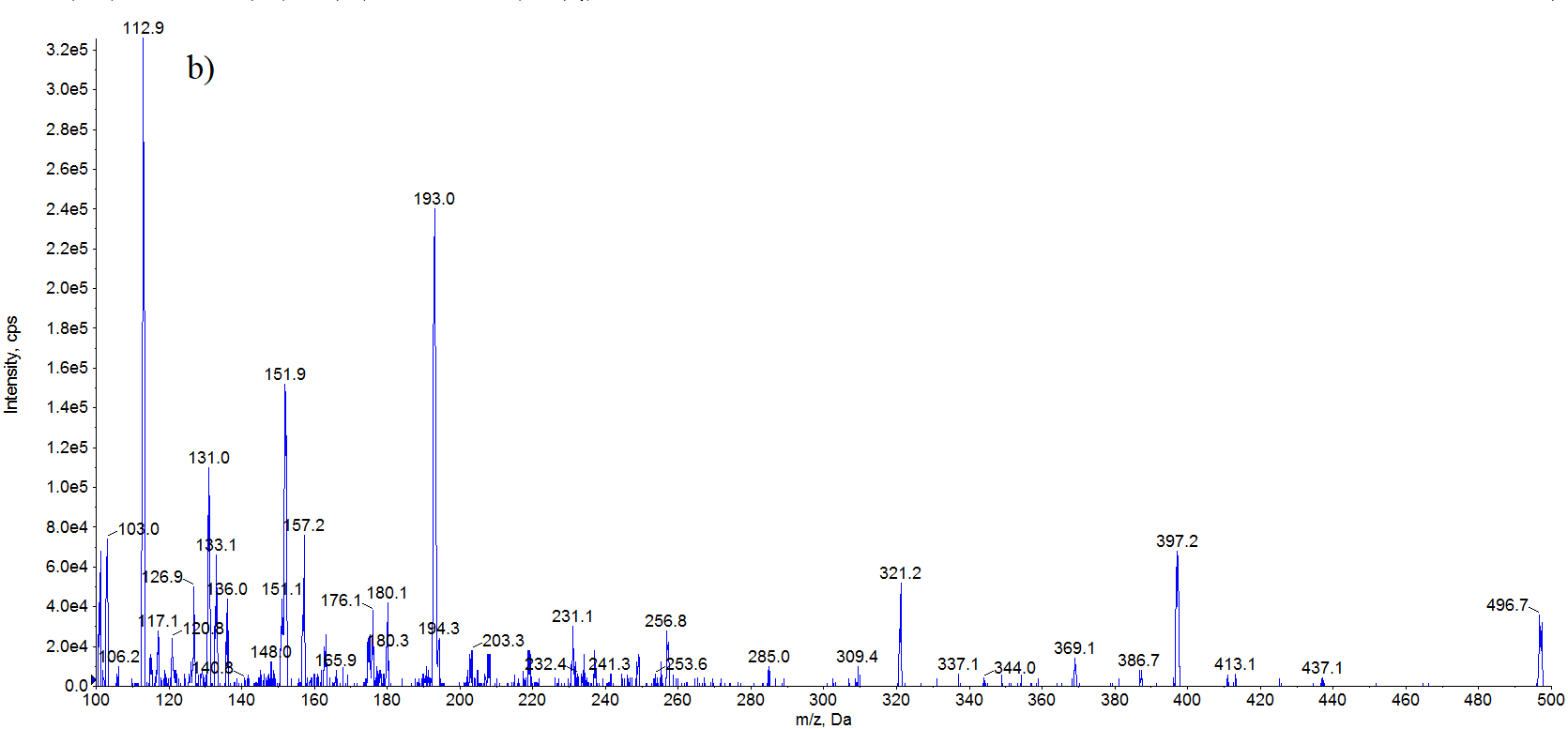

Supplement: Supplementary file 1 [file animals-10-00030-s001.zip › Supplementary Materials/Supplement 3.tif]

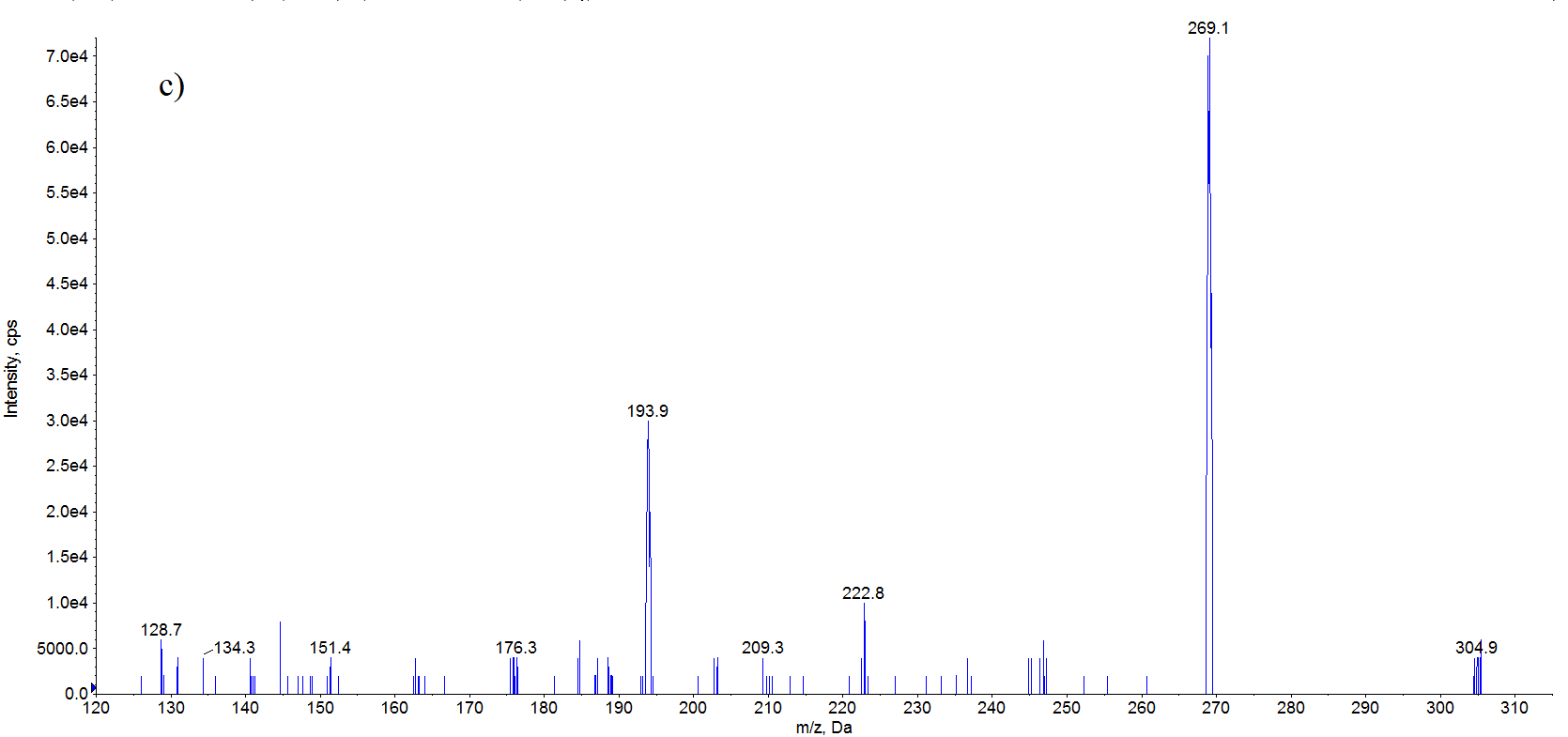

Supplement: Supplementary file 1 [file animals-10-00030-s001.zip › Supplementary Materials/Supplement 4.tif]

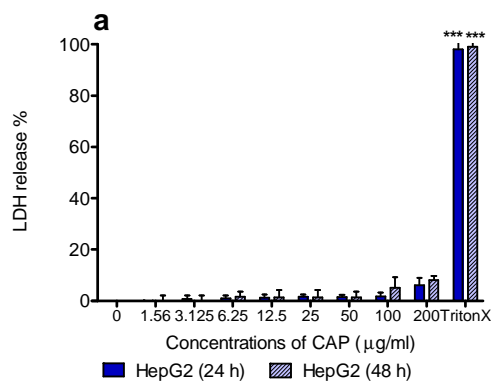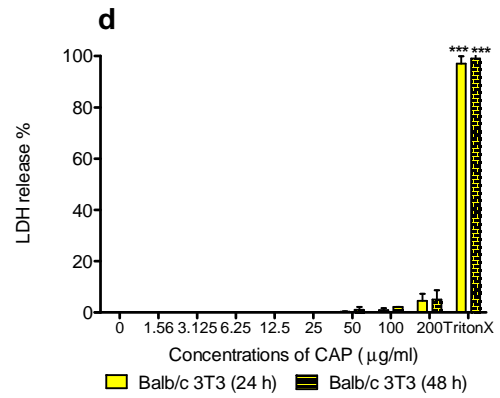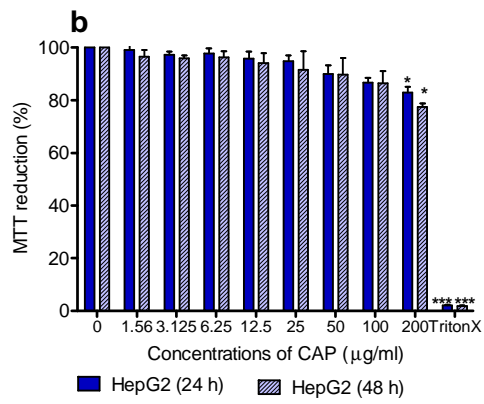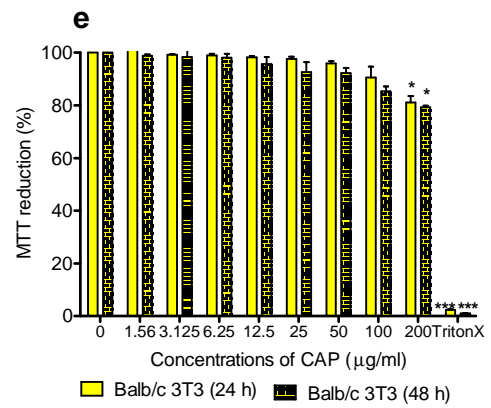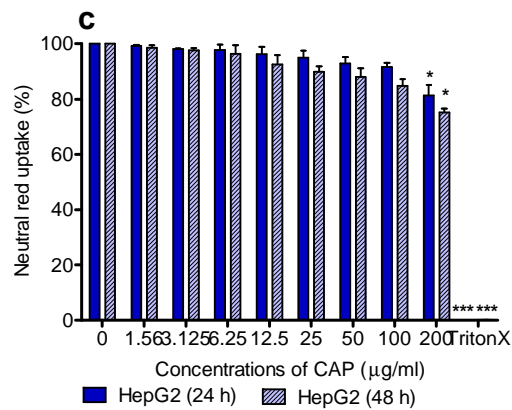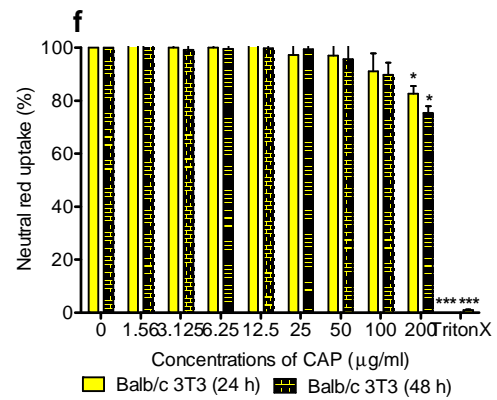

Supplement: Supplementary file 1 [file animals-10-00030-s001.zip › Supplementary Materials/Supplement 5.pdf]

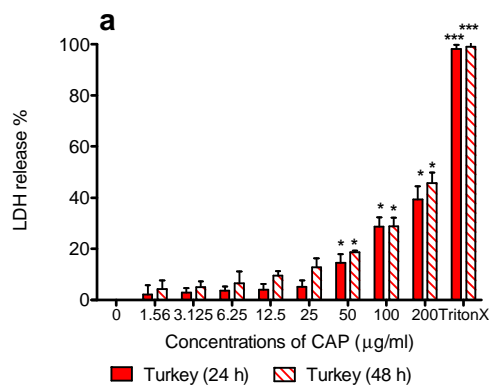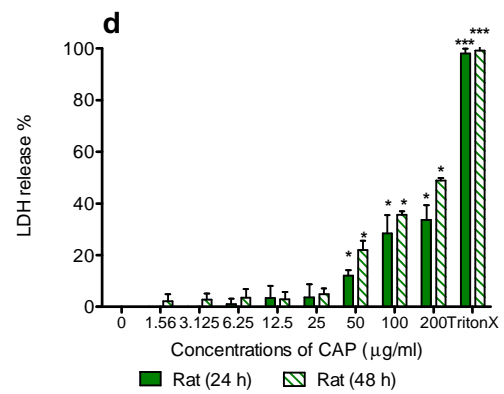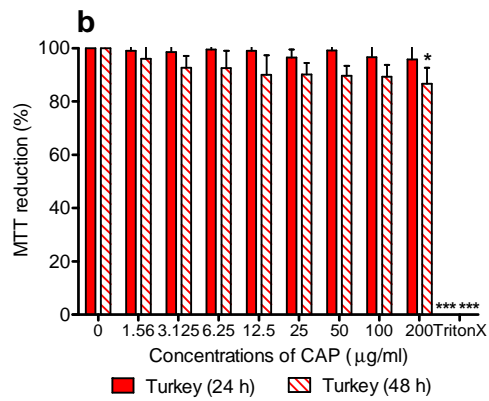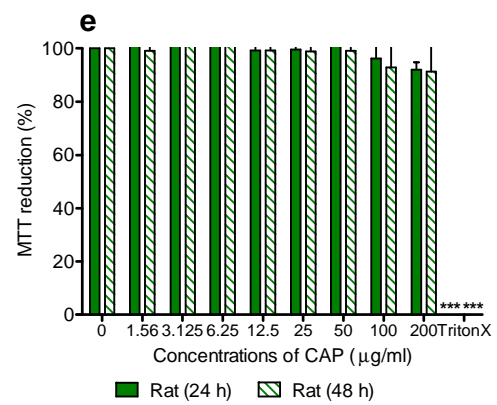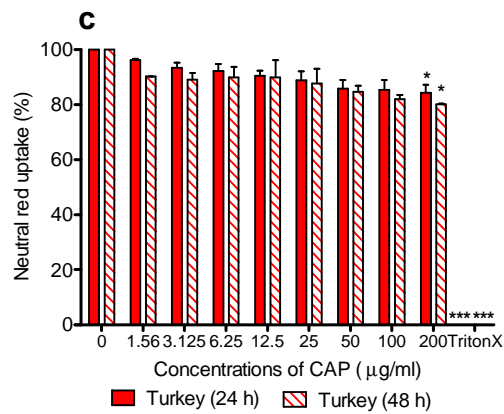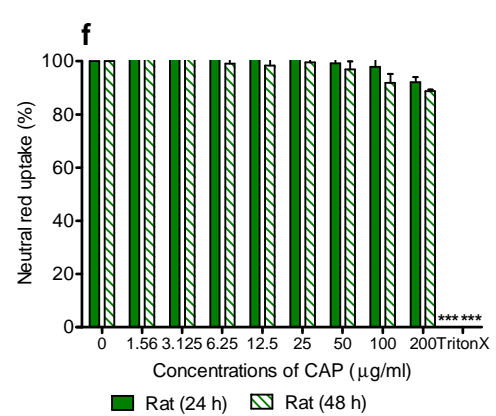

Supplement: Supplementary file 1 [file animals-10-00030-s001.zip › Supplementary Materials/Supplement 6.pdf]

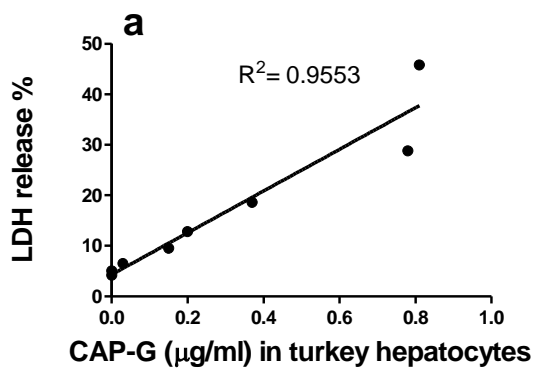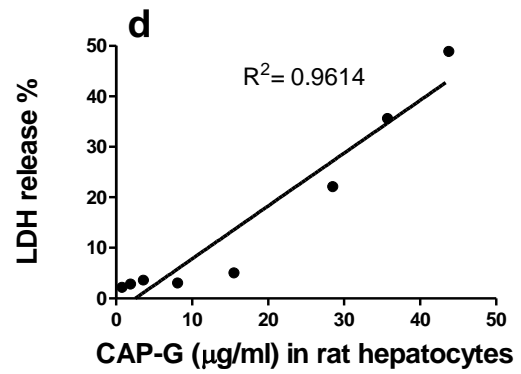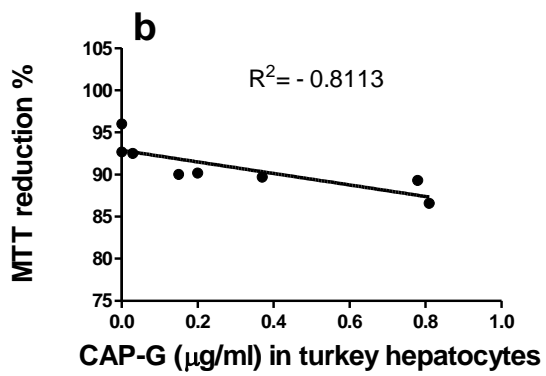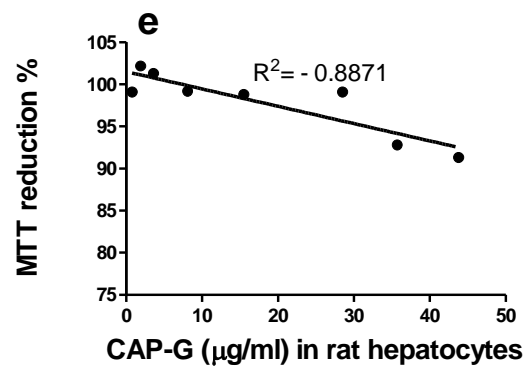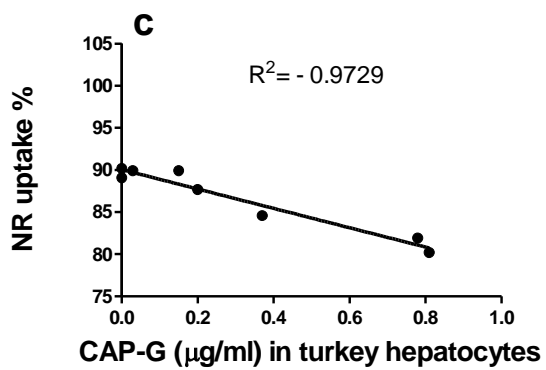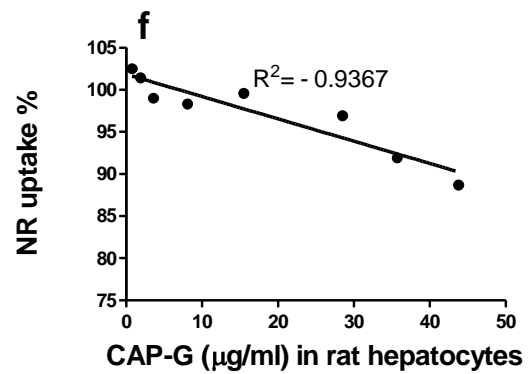

Supplement: Supplementary file 1 [file animals-10-00030-s001.zip › Supplementary Materials/Supplement 7.pdf]
